# Effect of Climate Change on West Nile Virus Transmission in Italy: A Systematic Review

**DOI:** 10.3389/phrs.2025.1607444

**Published:** 2025-04-25

**Authors:** Antonio Lorenzon, Michele Granata, Pietro Verzelloni, Luigi Tommasi, Lucia Palandri, Marcella Malavolti, Annalisa Bargellini, Elena Righi, Marco Vinceti, Stefania Paduano, Tommaso Filippini

**Affiliations:** ^1^ Department of Biomedical, Metabolic and Neural Sciences, Section of Public Health, University of Modena and Reggio Emilia, Modena, Italy; ^3^ Department of Epidemiology, School of Public Health, Boston University, Boston, MA, United States; ^4^ School of Public Health, University of California, Berkeley, Berkeley, CA, United States

**Keywords:** arbovirus, *Culex pipiens*, climate change, mosquito, West Nile virus

## Abstract

**Objectives:**

West Nile Virus (WNV) infection prevalence is increasing in recent years in Europe, particularly in Italy. Such increase has been related to climate and environmental factors. Our review aims to assess the relation between climate change-related factors and the spread of WNV in Italy.

**Methods:**

We conducted a literature search across four online databases until 22 January 2025, using as search terms WNV, its vectors, and climate change.

**Results:**

Out of 282 unique articles, we included 29 eligible papers published between 2011–2025, most of them assessing distribution of the main WNV vector (*Culex pipiens*) and epidemiology of the infection in relation to climate/environmental factors. We found a positive strong association of WNV transmission with temperature and agricultural land use. Associations with other environmental variables also emerged, but they were either weak or inconsistent.

**Conclusion:**

Despite some inconsistencies in the results, likely due to heterogeneity in study methodologies and interactions of environmental variables, review findings indicate that some climate change-related factors favor WNV spread through its vectors in Italy, in line with exploratory observations obtained on the entire Europe.

**Systematic Review Registration:**

https://www.crd.york.ac.uk/PROSPERO/view/CRD42023430636, identifier CRD42023430636.

## Introduction

West Nile fever is a disease originating in subtropical areas, which in recent decades has been increasing in Europe, especially in Southern Europe [1], but there are also concerns about its spread to other parts of the continent [2]. West Nile disease is an arbovirosis, i.e., an infection caused by viruses transmitted by vectors, represented in Europe mainly by two mosquitoes of the genus *Culex* (*Cx.*), *Cx. pipiens* and *Cx. modestus* [3]. The reservoirs of this pathogen are birds, especially magpies, grey crows, turtle doves and passerines, who live in both natural and urban environments, and are all characterised by large migrations. For this latter reason, the virus spreads through the vectors and infects other hosts, especially but not limited to equines and humans, affecting also reptiles, amphibians and other mammal species [4–8]. In humans, the infection runs asymptomatically in most cases, but 20%–30% of subjects present the symptomatic form, i.e., West Nile fever. About 3% of symptomatic cases and 0.5%–1% of total cases present a neuroinvasive form possibly leading to serious consequences, up to death, in about 10% of cases [9–11].

Before 1998, there were only few documented cases of West Nile Virus (WNV) in Europe, and none in Italy [12]. Since then, the disease has dramatically increased, leading to 475 human neuroinvasive forms in Italy alone during the decade 2008–2018. This surge has shown no sign of curbing: in 2018, 2083 cases and 180 deaths were recorded in Europe, 577 and 46 of them respectively in Italy [13, 14]. In the 2024 transmission season, Italy has reported 455 locally acquired human cases (of 1436 in Europe) and 21 deaths (of 125 in Europe) related to WNV infection, thus making it the most affected European country for several years already, followed by Greece (217 cases with 34 deaths) [15].

Public attention is increasingly focusing on this spread as its causes are still unknown, although climatic and environmental factors are prime suspects, as observed for other infectious diseases [16–19]. One of the major suspected causes is climate change [20]. As a matter of fact, similar trends have been noted between the increasing impact and severity of climate change and the WNV epidemics in the same territories [21]. It has been shown that the increased spread of vectors in European countries has been caused also by the increase in temperatures linked to global warming, which has expanded suitable areas and lengthened the survival period of the vectors [22–24]. Although some evidence on the association between climate change and WNV spread has been recently reported, literature on a comprehensive overview and comparison of the climatic and environmental factors involved is missing. Some recent reviews have approached the topic in a rather exploratory way, including studies referring to a large time interval to extremely different territories and in some cases considering several arboviruses together. Also because of the low comparability of the data, they were limited to describing the presence or absence of associations, without going into detail [25, 26].

This systematic review aims to analyse the existing epidemiologic evidence investigating the association between the spread of WNV or its vectors, and climatic or environmental factors, focusing on Italy as the most affected country in Europe [15].

## Methods

### Review Registration and Research Question

We performed a systematic review of the literature on the association between climate and environmental factors and WNV and its vectors epidemiology in Italy. The recommendations of the PRISMA (The Preferred Reporting Items for Systematic reviews and Meta-Analyses) guidelines were followed and the study protocol was registered in PROSPERO (no. CRD42023430636) [27].

The research question following the PECOS (Population, Exposure, Comparison, Outcome, Study design) statement was “What is the association between climatic or environmental factors and WNV infection prevalence/WNV vectors distribution in Italy?”. Inclusion criteria were as follows: Italian human and non-human population (P), climatic and environmental factors associated with climate change (E), comparison between such factors, i.e., low or no exposure (C), increase in WNV cases or vectors spread (O) and non-experimental and experimental studies (S).

### Literature Search and Screening

We searched four literature databases (PubMed, Web of Science, Embase, and Scopus) up to 22 January 2025, using MeSH terms related to WNV and its vectors (*Cx. pipiens* and *modestus*) as reported in detail in Supplementary Table S1. We considered only original research studies with qualitative or quantitative analysis in English or Italian language, and we also screened the references lists of the retrieved articles for additional eligible studies. Two of us (AL and PV) independently screened the title, abstract and full text of the studies. We used the freely available systematic review tool Rayyan^1^ for listing the selected papers and references retrieved were saved in Endnote (Clarivate Analytics, 2024).

### Inclusion and Exclusion Criteria

We considered as eligible for our review only original studies assessing the geographical distribution of vectors or WNV infection in humans or non-human hosts in Italy, also considering climatic, environmental, or geographical factors (Supplementary Material on the selection process). We did not include case studies and literature reviews, other reports and guides published by relevant organizations, unpublished manuscripts, conferences proceedings, notification of outbreaks, and clinical descriptions of diseases.

### Data Extraction

From the selected articles, we extracted data about first author, publication year, study design, region of the study, vector species, reservoir and hosts (humans, non-human hosts–i.e., birds or other animals - and bird species), type of data presentation (e.g., abundance or probability map), analytical approach, data sources, climatic and environmental factors, and WNV activity indicator. Both vector and WNV related outcomes were considered as WNV activity indicators. Vector related outcomes included abundance, expansion, growth rate, infection rate and geographical suitability for mosquitos. WNV related outcomes included abundance, incidence, force of infection (FOI), spillover risk, circulation, and transmission rates of the virus.

### Quality Assessment

To evaluate quality of the selected publications, a 12-items quality assessment tool was used and adapted, based on methods applied in similar reviews [26]. Quality was scored as a binary variable (yes/no scoring as 1/0). The sum of positive items yielded the final evaluation, with higher scores meaning higher quality (maximum score: 12). The items evaluated included: description of the most relevant background, clear description of the objectives, appropriate study design, description of data collection procedures, sources for data collection, description of the study period, description of the place of study, description of the analytical approach and methods, description of data analysis, clear description of the results, results coinciding with objectives, adequate conclusions.

### Data Analysis

In order to compare results, we implemented a graphical representation showing the number of studies investigating each factor and the direction (positive or negative) of the association emerged, if any. Each factor was assigned a specific circle. The size of the circle was weighted according to the number of studies that investigated that specific factor, while on the x-axis was the percentage of such studies in which the factor was found to be associated with the outcome. Furthermore, within the circle was the relative frequency, in percentage, in the sign of the associations found (grey for positive and black for negative). We used Mapchart^2^ and Excel (Office Package, Microsoft Corp., Redmond, WA, 2024) to produce tables, figures, and maps.

## Results

### Study Inclusion

**FIGURE 1:**
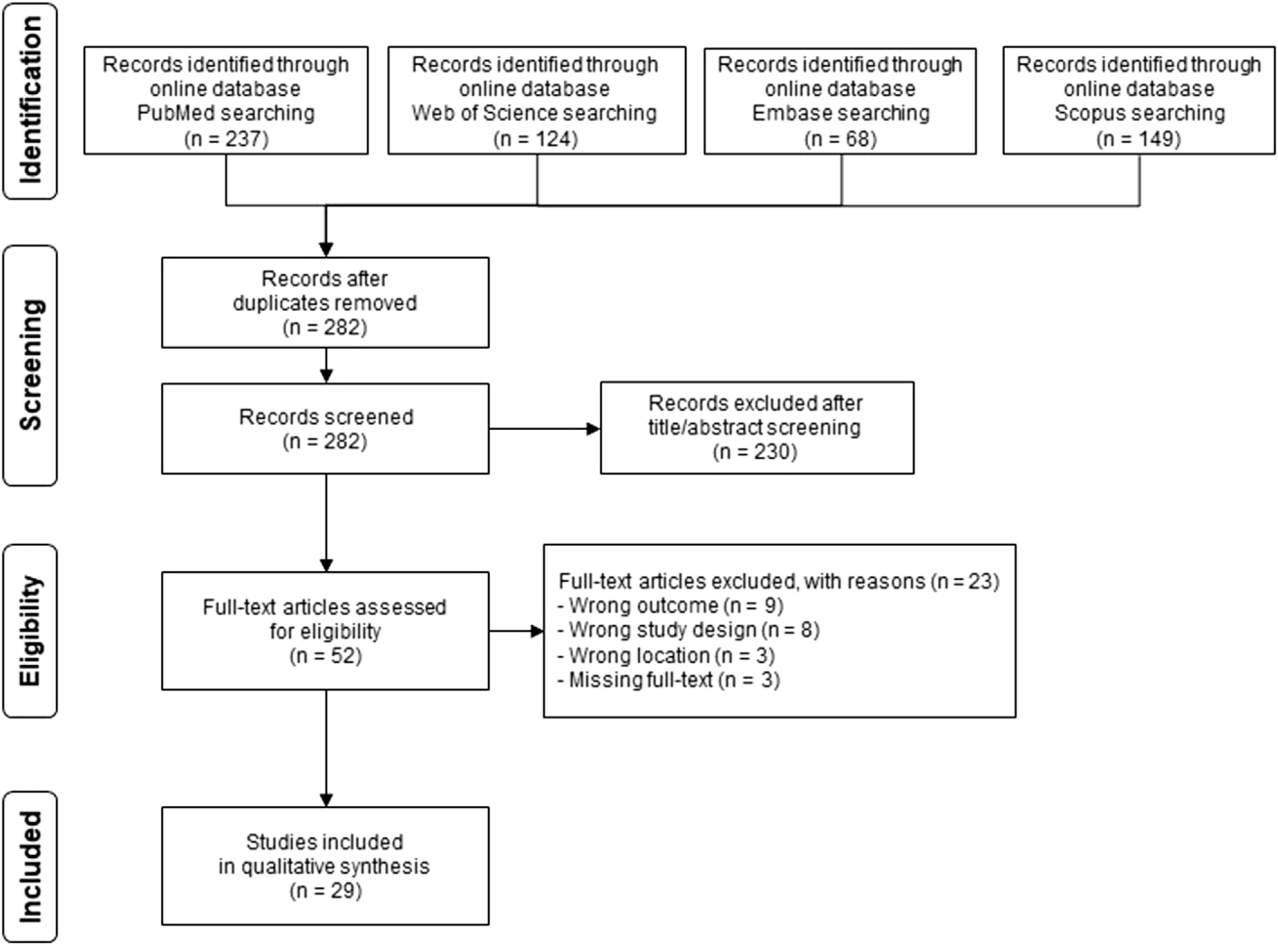
PRISMA flow chart of the inclusion process (systematic review, Italy, 2011–2025).

The database search yielded 578 potentially eligible articles (Figure 1). After exclusion of 296 duplicates, we performed title and abstract screening of the 282 remaining items. Out of 52 articles selected for full-text evaluation, we eventually included 29 papers in our review. Reasons for exclusions were an outcome unrelated to WNV or disease (n = 9), study design not reporting original data (n = 8), missing full text (n = 3) or not specifically considering Italy (n = 3), as reported in Figure 1.

### Study Characterization

The main characteristics of the studies included are summarised in Table 1. All articles were published between 2011 and 2025. The most frequently used statistical model types were association/correlation models (n = 14) followed by predictive models (n = 4). Other models were spatial models (n = 1), density/abundance models (n = 2), and multiple models (n = 8). Among the latter, generally spatial (n = 2) or transmission models (n = 4) were mixed with other types. Two studies mixed association and predictive models.

**TABLE 1:** Summary information of the studies selected for review (systematic review, Italy, 2011–2025).

General information				Host infected			WNV	Vector		Study place	
First author (year) [reference number]	Study design	Analytical approach^a^	Study quality	Human	Birds	Equines	Effect on WNV studied	Analyzed *Culex* species	Effect on vectors	Analyzed region	Type of maps^b^
Bisanzio et al. [28]	Ecological	SM/AM	9					both^c^	Abundance	Piedmont	AM, PM
Calzolari et al. [29]	Ecological	SM	11					*pipiens*	Vector infection	Emilia-Romagna, Lombardy	AM
Calzolari et al. [30]	Cross sectional	SM/DM	9					*pipiens*	Vector infection	Padan Plain	AM
Candeloro et al. [31]	Ecological	PM	9		X	X	WNV circulation			Italy	PM
Carrieri et al. [32]	Longitudinal	AM	11					*pipiens*	Abundance	Bologna Province	
Conte et al. [33]	Ecological	AM/PM	10	X		X		both^c^	Suitability	Italy	PM
De Angelis et al. [34]	Case Time Series	AM	12	X			Incidence			Northern Italy	AM
De Freitas Costa et al. [35]	Ecological	TM/DM	11		X		Birds Transmission	both^b^		Italy	
Fesce et al. [36]	Ecological	TM/PM	10		X		Birds Transmission	*pipiens*	Vector infection	Lombardy	
Fornasiero et al. [37]	Ecological	AM	12					both^c^	Growth rate, Abundance	Veneto, FVG^d^	
Groen et al. [38]	Cross sectional	PM	8					*pipiens*	Abundance	Emilia-Romagna, Po Delta	
Jian et al. [39]	Ecological	AM	10					*pipiens*	Growth rate, Abundance	Rovigo Province	
Marcantonio et al. [40]	Ecological	AM/PM	11	X			Incidence			All Europe	AM
Marini et al. [41]	Longitudinal	DM	10					*pipiens*	Abundance	Eastern Piedmont	
Marini et al. [42]	Case control	DM	10					*pipiens*	Abundance	Trento and Belluno Provinces	
Marini et al. [43]	Ecological	DM/TM	9	X	X		Spillover Risk	*pipiens*	Abundance	Veneto	
Marini et al. [44]	Ecological	TM/AM	9				Birds Transmission	*pipiens*	Abundance	Emilia-Romagna	AM
Marini et al. [45]	Ecological	AM	10	X			Abundance			All Europe	AM
Marini et al. [46]	Ecological	AM	8	X			Force of infection			All Europe	AM
Moirano et al. [47]	Ecological	AM	11	X			Incidence			Northern Italy	AM
Moirano et al. [48]	Case Crossover	AM	10	X			Incidence			All Europe	AM
Mughini-Gras et al. [49]	Ecological	PM	10			X	Abundance			Italy	AM, PM
Mulatti et al. [50]	Ecological	AM	8		X	X	Abundance			Padua, Rovigo, Venice Provinces	PM
Mulatti et al. [51]	Longitudinal	AM	10					*pipiens*	Expansion	Veneto, FVG^d^	AM
Rizzoli et al. [52]	Cross sectional	AM	12		X			*pipiens*	Abundance	Veneto	
Roiz et al. [53]	Cross sectional	AM	11					*pipiens*	Abundance	Trentino (Arco, Riva del Garda)	
Rosà et al. [54]	Ecological	AM	11					*pipiens*	Abundance	Piedmont	
Serres et al. [55]	Ecological	PM	12	X			Outbreaks			All Europe	PM
Trajer et al. [56]	Ecological	AM	10	X			Abundance			Italy	

^a^ Analytical approach: predictive models (PM), association models (AM), spatial models (SM), transmission models (TM).

^b^ Type of maps: abundance map (AM), predictive map (PM).

^c^ Both *Cx. pipiens* and *modestus*.

^d^ FVG (Friuli-Venezia Giulia).

Considering quality assessment, the average score was 10 points. The most frequent gaps in the score were related to a lack of clear and complete description of the objectives (11 studies) and of the results (10 studies), while 6 studies did not accurately report the study or data collection period and five did not have exhaustive conclusions (Supplementary Table S2).

The analysed outcome was WNV in 12 studies, the vector in 14, while 3 studies assessed both endpoints. Of those related to WNV, 2 studies analysed the total number of cases in humans [45, 56], one study in horses [49] and one study in horses, birds and mosquitoes [50]. Four studies evaluated the incidence of WNV infection in humans [34, 40, 47, 48], and 3 others viral transmission between mosquitoes and birds [35, 36, 44]; one study the circulation of the virus in animals [31], the FOI [46], the spillover risk [43] and outbreaks [55].

In contrast, the studies that reported the spread of the vector as an outcome analysed absolute abundance in 10 studies [28, 32, 38, 41–44, 52–54], abundance together with growth rate in 2 studies [37, 39], the percentage of vector infection in 3 studies [29, 30, 36], and expansion [51] and geographical suitability [33] in one study each.

**FIGURE 2:**
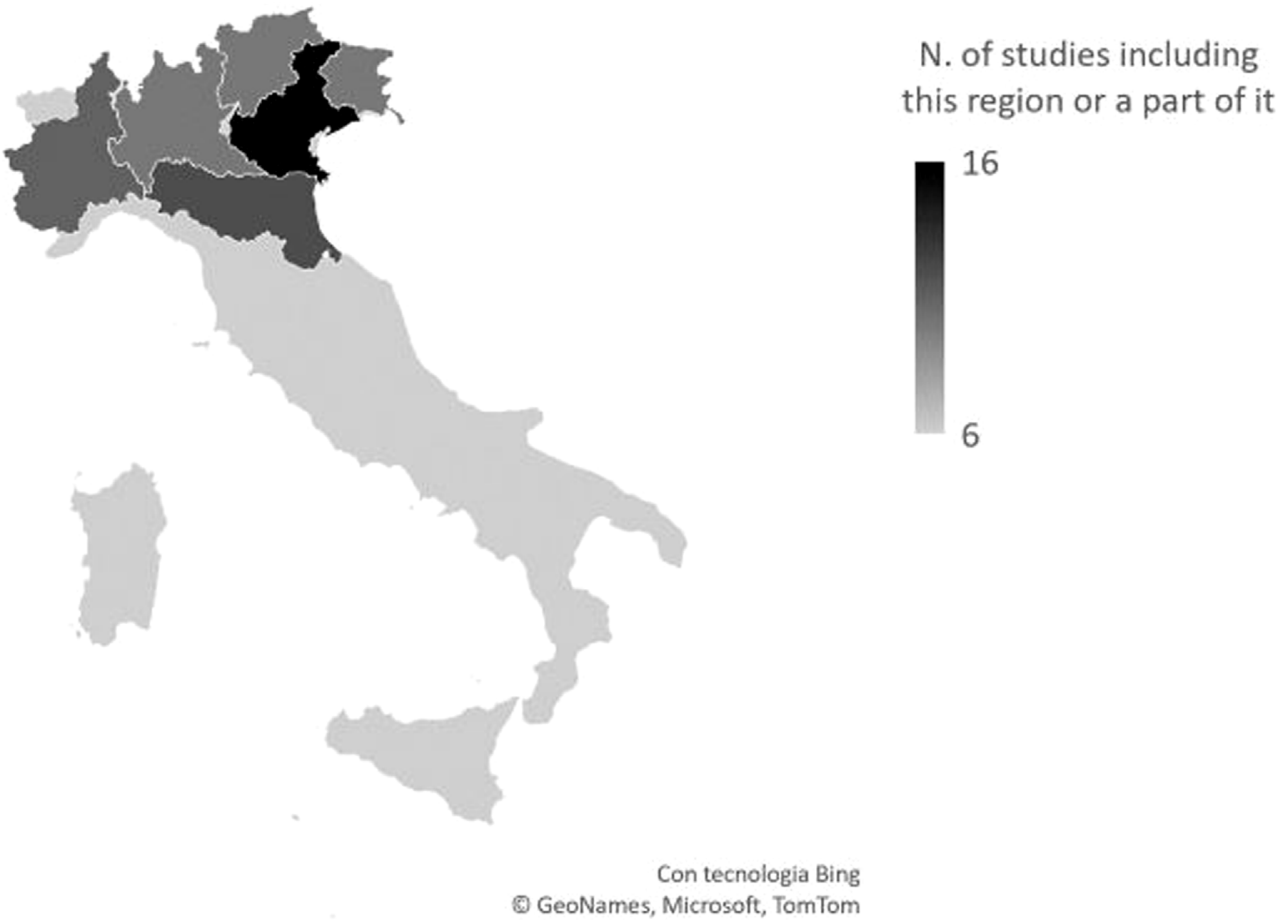
Geographical distribution of studies. The whole Italian territory was considered in six works (systematic review, Italy, 2011–2025).

As depicted also in Figure 2, eight studies analysed the entire country, and 3 studies Northern Italy only. The remaining 16 studies focused on specific Northern Italy areas, namely, 7 studies the so-called Triveneto (Friuli-Venezia Giulia, Veneto, Trentino), 2 studies Emilia-Romagna, and 2 studies both Emilia Romagna and Triveneto. Finally, 3 and 2 studies assessed the Piedmont and the Lombardy region, respectively.

### Climatic and Environmental Factors

**FIGURE 3:**
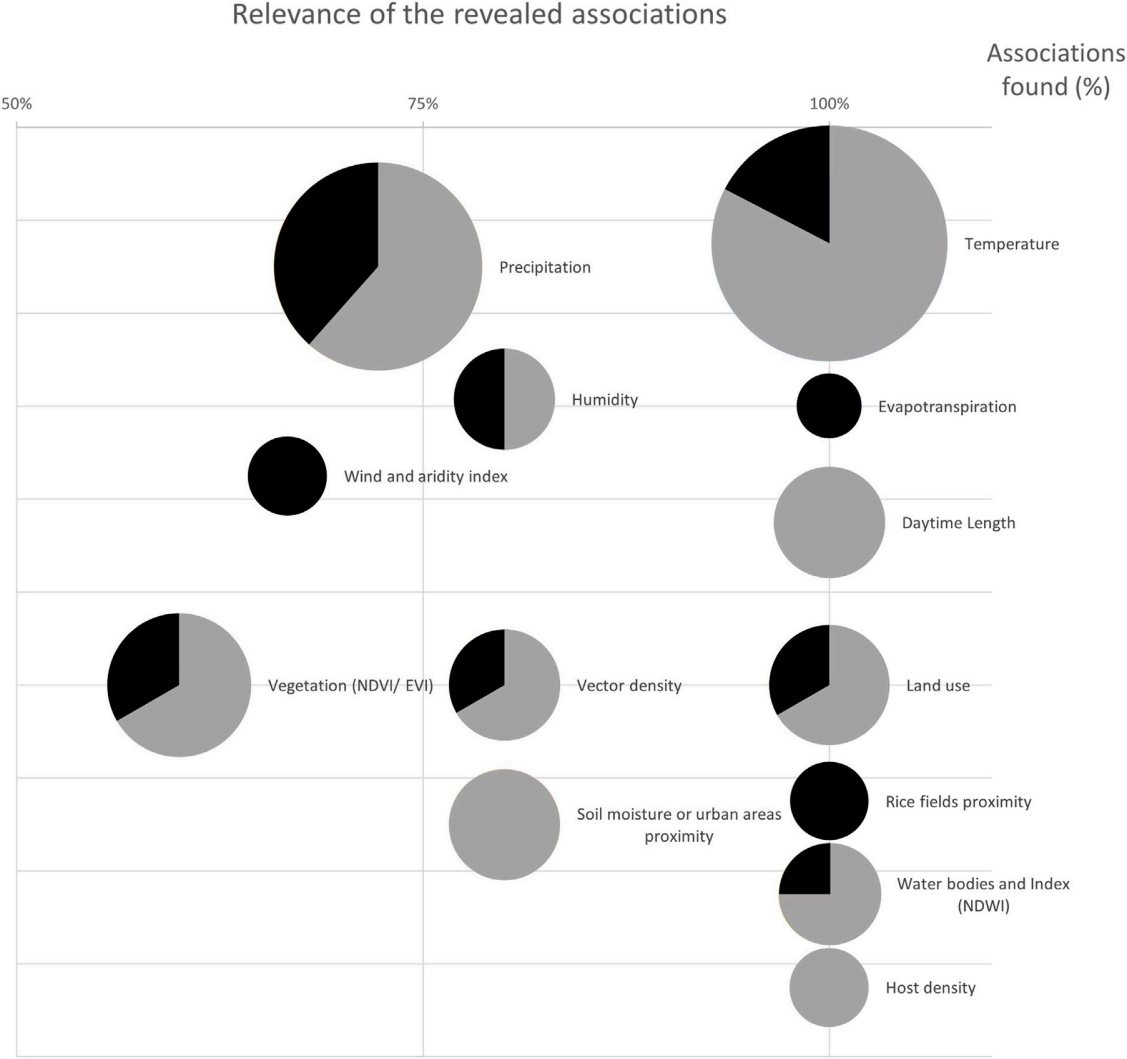
Graphical summary of the emerged associations. The size of the circles represents the number of studies evaluating each factor, while the x-axis shows the percentage of times an association emerged (100% means that an association was found in all the studies). Within the circle is the proportion in the sign of associations: grey for positive, black for negative (systematic review, Italy, 2011–2025).

Associations of WNV transmission and vector epidemiology with climate and environmental factors emerged in the studies, are described below (Figure 3; Supplementary Tables S3, S4), as follows:

#### Temperature

Temperature was the most investigated factor as it was considered in 27 studies. In many of these it proved to be a key factor in the spread of WNV and its vector, with a high rank in predictive models [28, 31, 49, 50] and showing almost always positive associations (24 out of 27 studies). Only 3 studies reported a negative association between temperature and vector abundance, 2 of which were related to the rise in temperature in the preceding days [37, 51]. Conversely, one study reported the competition with mosquitoes of the genus *Aedes* (*Ae.*), which was greater at higher temperatures [42]. All other associations that emerged were positive. In particular, positive associations between the average temperature of a preceding period of 1 week or more, up to more than a month, and abundance of the vector, infection rate of the vector or incidence of WNV in the population were found [29, 34, 38, 47, 48, 53, 56].

High spring temperatures have been shown to anticipate the first seasonal WNV cases, to lengthen the epidemic season and to be positively associated with vector or WNV abundance in summer, or with FOI [32, 41, 44–46, 54]. The mean temperature during summer months positively correlated with vector abundance and disease incidence [32, 40]. In one paper, summer temperatures ranging from 15°C to 28°C were positively associated with the occurrence of WNW cases in humans [55]. A predictive model showed that the warmer temperature in Italy compared to other European countries are associated with viral circulation despite the low vector-host ratios (number of mosquitoes per bird), and the high dilutions, i.e., high proportion of mosquito bites on competent birds that are able to replicate the virus to infect mosquitoes [35]. In another study a negative association emerged between late summer heat and vector abundance, but with a longer period of vector activity [54].

Temperature was found to be, on average, higher in WNV endemic areas than in neighboring areas, and a predictor of the risk of outbreaks [30, 36, 55]. Finally, collinearity emerged with both mean day length [39] and humidity [38].

#### Precipitation

Rainfall was evaluated in 21 studies, with conflicting results. In 5 of these, rainy days or the precipitation amount were not associated with expansion, growth, abundance, or infection rate of the vector, nor with FOI [29, 37, 39, 46, 51]. In other studies, however, a positive association emerged between heavy rainfall in the previous month and WNV incidence [47]; overall a positive association or predictive value was found in 5 other studies [32, 40, 41, 48, 55]. According to one study, rainfall decreased competition with *Ae. albopictus* mosquitoes, increasing the number of *Cx. pipiens* [42]. In 4 studies, spring precipitation was positively associated with vector abundance during the summer season, although it may delay its onset and shorten its duration [32, 40, 41, 54]. Summer rains, on the other hand, were associated with outbreaks probability, a greater presence of vectors in the following months and a longer duration of the epidemic [32, 54, 55]. Finally, according to 4 studies intense rainfall was negatively associated with both the abundance of the vector up to 7 weeks later, and the monthly cases amount; furthermore, rain was found to be lighter in virus endemic areas than in neighboring areas [30, 34, 38, 53, 56].

#### Humidity

Humidity was evaluated in 5 studies, resulting not associated with vector expansion in one of them [51]. Negative associations were found with vector abundance 3–4 weeks later [38] and with incidence of WNV infection in humans 1–9 weeks later [34]. In other research, high relative humidity in May, July and August was positively associated with summer abundance of the vector [32] and relative humidity levels from 70% to 85% positively influenced the probability of occurrence in human cases of WNV infection [55].

#### Wind

The role of wind was assessed in 2 studies, being not associated with vector abundance in one study [39], while negatively correlated towards it from May to August in the other one [32].

#### Evapotranspiration

Evapotranspiration is the loss of water from the soil both by evaporation from the land surface and by transpiration from the plants growing on it [57]. It was evaluated by 2 studies: one study reported a negative association between evapotranspiration in May and vector abundance in the subsequent first three summer months, while the other one pointed out a strong negative association with vector infection rate [30, 32].

#### Daytime Length

Daytime length was positively associated with vector abundance, expansion and spillover risk in all the 6 studies that analysed it [35, 37, 39, 40, 43, 51].

#### Thermal Excursion and Hydroclimatic Balance

These two factors were both analysed once. Thermal excursion, particularly that of April and May, was found to be negatively associated with vector abundance in the early summer period. In contrast, the hydroclimatic balance (calculated as rainfall minus evapotranspiration) was positively associated with the abundance of the vector from May to September [32].

#### Vegetation Index (NDVI/EVI)

The Normalized Difference Vegetation Index (NDVI) is a satellite-based measure calculated from the near infrared and visible wavelength values reflected by the plants. It is indicative of vegetation density and values range from −1, corresponding to area with no or low vegetation like water bodies or areas covered by cement, and +1 corresponding to high density vegetation like forest; the Enhanced Vegetation Index (EVI) is similarly calculated, but it corrects for some distortions in the reflected light caused by the particles in the air as well as the ground cover below the vegetation [58]. Vegetation index was evaluated in 10 studies [28, 30, 31, 33, 37, 39, 40, 49, 50, 55], 2 of which observed a positive association with vector growth and abundance [37, 39]. Two studies concluded that there was no association between vegetation and WNV incidence [40] or outbreaks [55], while another observed more dense vegetation outside the area of virus circulation [30].

#### Presence of Standing Water or Wetlands

The presence of standing water was analysed in 2 studies [40, 55]. One of these concluded that it was not a predictor of virus incidence [40] and another assessed that the presence of open water areas, wet crops or wetlands was a positive predictor for outbreaks [55].

#### Water Index (NDWI) or Aridity (De Martonne Index)

The normalised difference water index (NDWI) is used to delineate open water features and enhance their presence in remotely-sensed digital imagery [59]. It was analysed in 2 studies and was found to be positively associated with earlier onset and longer duration of seasonal vector presence [54], while its spring and summer values were negatively associated with WNV incidence [40]. Only one study assessed aridity (De Martonne aridity Index, calculated as the ratio between the annual average precipitation and the annual average temperature increased by 10°C [60]) as a function of vector abundance, showing a negative association [37].

#### Altitude

The role of altitude was a considered in 4 studies [28, 33, 49, 50], of which only one analysed the individual contribution of this variable, defining it as very important in delineating a suitability area for the vector [33].

#### Human Use of the Environment (Land Use)

Human land use was taken into account 5 times [28, 40, 46, 49, 55], being once negatively associated with FOI [46]. In other studies inhabited forests and more so irrigated croplands were found to be positive predictors for WNV incidence [40] and urban areas were positive predictors for outbreaks [55]. Furthermore, the presence of areas untouched by humans (such as protected areas) was not associated with vector abundance [52] and agroforestry areas, arable lands or arid shrublands were not predictive for the probability of outbreaks [55]. In the last study a set of variables evaluated through Corine Land Cover map was used to characterise the distribution of mosquitoes, but without defining which of them contributed most in predicting their presence [49].

#### Host Density

Host density, animal or human, was used as a variable in 3 studies [46, 49, 55], with 2 of them reporting a positive association: between density of individuals over 65 and FOI [46], and between human population density and probability of outbreaks [55].

#### Distance to Specific Areas

Emerging as non-predictive on FOI in one study [46], distance to specific areas was analysed in 4 other studies by:

a. Distance from rice fields [28, 39, 54], resulting having a negative association with vector abundance in all 3 studies.
b. Distance from damp soil/soil moisture [31, 39, 55], resulting in a negative association with vector abundance [39] and a high predictive value 80 days before WNV circulation [31]. The level of soil moisture has a positive predictive role for probability of outbreaks [55].
c. Distance from urban areas [28], negatively associated with vector abundance for *Cx. pipiens.*
d. Distance from forest areas [55], positive predictor for probability of outbreaks.
e. Distance from pastures [55], not predictive for outbreaks.

#### Other (Vector and Infection Dynamics)

Vector density, analysed in 7 studies, outlined 3 positive associations with consequently vector abundance, with transmission of WNV from birds to humans, or from mosquitoes to birds [32, 35, 41, 44], one non-linear association with mosquito positivity for WNV when tested by real-time PCR [29] and two negative associations with vector expansion after the reaching of a ceiling effect [37, 51]. WNV incidence in previous years was not associated with FOI [46]. In contrast, competition with *Aedes* mosquitoes was negatively associated with *Cx. pipiens* abundance [42]. Finally, infection dynamics in birds appeared to have a high impact on transmission to humans [36].

## Discussion

The most widely evaluated factors were temperature and precipitation (in 27 and 21 studies respectively), although consistent results were reported for the former only. An association between temperature and the spread of WNV or its vector emerged in 100% of the studies, being positive in 83% of them. Conversely, precipitation provided evidence of any association in only 2 out of 3 studies, with a positive direction in 62% of them and a negative direction in 38%. Environmental factors linked to the water cycle and humidity gave more contrasting results as the associations were generally not consistent, although the limited number of studies hamper any definitive conclusion. Conversely, greater consistency emerged from climatic factors such as wind, aridity, evapotranspiration, generally negatively associated, and hydroclimatic balance positively, with the outcomes.

Vegetation index was the third most frequently studied factor, being associated in 67% of the included studies to one of the outcomes of interest, positively with vector abundance or growth rate, and negatively or not associated with WNV incidence, presumably also due to fewer people in these areas. In contrast, WNV spread was positively associated with human use of the environment (irrigated croplands and settled forests) [40], which was instead negatively associated with FOI [46]. On the other hand, soil exempt from human use was not associated with vector abundance [52]. Moreover, distance from rice fields, damp soils and urban areas were found to be negatively associated with vector abundance [28, 39, 54], and the density of over 65 inhabitants positively with FOI [46]; all signs that human activities close to suitable areas have an impact on disease dynamics.

Finally, day length was found to be positively associated with outcomes, effectively confirming their seasonality, while temperature excursion negatively. Vectors and transmission dynamics appeared to be associated in a complex and non-linear manner with the spread of infection [35, 36, 42].

Our results are somewhat consistent with previous reviews that showed a high heterogeneity of the factors evaluated as well as of their associations with WNV [25, 26]. Conversely, we confirmed a strong positive association of temperature (especially at the onset of the warm season), of daylight hours and of anthropogenic impact with the spread of WNV and its vectors. In addition, our results further highlight that strong relevance of temperature in the spread of WNV and its vector. Thus, temperature increase linked to global warming in recent decades is expected to grow the number of cases of WNV in Europe and particularly in Italy. This effect does not seem to be related to the temperature peaks typical of the warmer months, but rather to the overall increase in its average values as well as to its reduced variation over days and seasons, yielding to the increase of both mosquitos and WNV presence [22–24]. These climatic modifications in Italy are already particularly evident and quickly escalating, accompanied by a sharp increase in cases, as shown in recent studies [61].

The relatively little influence of water cycle factors on the emerged outcomes can be explained in several ways. Indeed, the abundance of rainfall can lower the temperature, delaying the onset of the infectious season, and discourage both humans from going outside and mosquitoes from circulating, thus lowering the probability of contact. Conversely, dry periods accompanied by a scarcity of natural standing waters may push vectors, and birds, towards anthropogenic bodies of water close to inhabited areas, and paradoxically increase contagions as a result, as happened in the dry season of 2022 [62]. On the other hand, the abundance of rainfall in the warm months, and very full reserves from the spring rains, may increase the vector’s breeding sites, lengthening the season and increasing its abundance and WNV outbreaks probability [32, 54, 55].

However, rain and other factors linked to the water cycle have an overall contrasting effect. The region where WNV most affects Italy corresponds to the Padan Plain, a humid area with numerous watercourses and high human activity. Thus, abundant rainfall is not necessary to provide suitable breeding sites for *Culex* mosquitos. This could also explain why there is such a high disparity of WNV cases between the north and the south of the country. Southern regions are characterised by generally warmer temperatures and a more arid, uninhabited and windy landscape, all factors negatively associated with the outcomes considered [63].

The high level of human activity in northern Italy seems to be another relevant factor for the increase of WNV in Italy. In fact, while purely natural sites, where birds and watercourses abound, are positively associated with the presence of the vector, but negatively or not associated with the spread of WNV, those partially used by humans are positively associated with the spread of the virus, although negatively with FOI. The latter discrepancy could be explained by the fact that agricultural areas or settlements close to forests are places with a low population density. Indeed, the density of inhabitants over 65 years of age is positively associated with FOI, also due to their greater susceptibility to infection [64]. However, the presence of anthropic land use is a determining factor for the spread of WNV, the occurrence of which increases when approaching rice fields, urban areas and wet soils.

### Limitations and Strengths

The main limitations of this review concern the lack of uniformity in the selected studies. Indeed, these differ widely, both in terms of exposure and outcome variables collected, and even more so in terms of types of data analyses used. The wide variability of the studies constitutes a major impediment to the quantitative comparison and meta-analysis of the results, but sometimes even to the qualitative one, making them even inconsistent with each other in some cases. In addition, the low use of certain variables limits the interpretation of their effect, just as the inclusion of variables differing in quantity and type in the predictive models makes them difficult to compare with each other. In the future, it is hoped that evidence coming from upcoming literature will lead to more uniformity in the variable considered for each factor, so that results can be best compared with each other. Despite the poor comparability of the studies, results highlighted by this review are promising and already manage to outline an association between factors related to climate warming, anthropization of natural places, and the spread of WNV, along the lines of already existing reviews [25, 26]. This work, however, by focusing on a smaller but highly representative area, succeeds in going into greater detail on the variables analysed, providing new and more concise evidence, thereby providing important contributions to existing knowledge.

## Conclusion

In conclusion, climatic and environmental factors contribute through complex interactions of different intensity to the spread of WNV and its vectors. Global warming appears to be a determining factor for the recent sharp increase in cases in Northern Italy, through both the increase in average temperatures and the lengthening of the epidemic season. Factors linked to the water cycle seem to have little influence in an area characterised by great human population density and both agricultural and industrial activities, where there is plenty of breeding grounds for the vector. All these characteristics are associated to a high incidence of WNV or vector abundance, especially in contact areas between human and natural environments where there is no shortage of reservoirs of the virus and moist soil. In coming years, particular attention should be paid to very hot spring seasons, a warning sign for intense summer epidemics, and to human activities near natural environments or wet crops. Consequently, the future impact of WNV infection will depend on both policies against global warming, and protection of natural environments aimed at reducing the human invasiveness towards them.

## Footnotes

1
https://www.rayyan.ai/

2
https://www.mapchart.net/
