# Supplementary material for: Effect of Climate Change on West Nile Virus Transmission in Italy: A Systematic Review
Source: Public Health Rev. 2025 Apr 25;46:1607444. doi: 10.3389/phrs.2025.1607444 (PMC12061676; doi:10.3389/phrs.2025.1607444)
Supplement: Supplementary file 1 [file DataSheet1.docx]

**Supplementary Material**

**Additional information on selection process** (systematic review, Italy, 2011-2025).

This first filtering was carried out by responding to the following questions:

1. Did the study include Italy or a number of countries including Italy? Yes No Not certain.

2. A. Does it study the vectors’ geographical distribution? Yes No Not certain.

B. Does it study WNV infection in humans? Yes No Not certain.

C. Does it study WNV infection in non-human hosts? Yes No Not certain.

3. A. Does the study consider the relationship with climatic factors? Yes No Not certain.

B. Does it consider the relationship with environmental factors? Yes No Not certain.

C. Does it consider the relationship with geographical factors? Yes No Not certain

The article was discarded if the answer to Question 1 was no or if, in the second and third blocks of questions, the article did not receive at least one “Yes” per block.

**Supplemental Table S1**. Search strategies in online databases (systematic review, Italy, 2011-2025).

| **Database** | **Search string** |
| --- | --- |
| PubMed | (West Nile OR culex pipiens OR culex modestus) AND (Italy) AND (climat* OR environment* OR temperature OR warm* OR meteo* OR rainfall OR humidity OR altitude) |
| Web of Science | (West Nile OR culex pipiens OR culex modestus) AND (Italy) AND (climat* OR environment* OR temperature OR warm* OR meteo* OR rainfall OR humidity OR altitude) (Title) or (West Nile OR culex pipiens OR culex modestus) AND (Italy) AND (climat* OR environment* OR temperature OR warm* OR meteo* OR rainfall OR humidity OR altitude) (Abstract) |
| Embase | italy:ti,ab,kw AND ('west nile virus':ti,ab,kw OR 'culex pipiens':ti,ab,kw OR 'culex modestus':ti,ab,kw) AND (climate:ti,ab,kw OR environment:ti,ab,kw OR temperature:ti,ab,kw OR heat:ti,ab,kw OR meteorology:ti,ab,kw OR rain:ti,ab,kw OR humidity:ti,ab,kw OR altitude:ti,ab,kw) |
| Scopus | (TITLE-ABS-KEY(((West AND Nile) OR (culex AND pipiens) OR (culex AND modestus)) AND (Italy) AND (climat* OR environment* OR temperature OR warm* OR meteo* OR rainfall OR humidity OR altitude)) |

**Supplementary Table S2.** Quality assessment of the included studies (systematic review, Italy, 2011-2025).

| **First author (year) [reference number]** | **Description of most relevant background** | **Clear description of objectives** | **Appropriate study design** | **Description of data collection procedures** | **Sources for data collection** | **Description of study period** | **Description of place of study** | **Description of analytical approach and methods** | **Description of data analysis** | **Clear description of results** | **Results coinciding with objectives** | **Adequate conclusions** | **Total** |
| --- | --- | --- | --- | --- | --- | --- | --- | --- | --- | --- | --- | --- | --- |
| Bisanzio  (2011) [43] | yes | no | yes | no | yes | yes | yes | yes | yes | no | yes | yes | 9 |
| Calzolari  (2015) [54] | yes | no | yes | yes | yes | yes | yes | yes | yes | yes | yes | yes | 11 |
| Calzolari  (2020) [53] | yes | no | yes | yes | yes | yes | yes | yes | yes | no | yes | no | 9 |
| Candeloro  (2020) [39] | yes | yes | yes | yes | yes | no | yes | yes | no | yes | yes | no | 9 |
| Carrieri  (2014) [44] | yes | yes | yes | yes | yes | yes | yes | no | yes | yes | yes | yes | 11 |
| Conte  (2015) [56] | yes | yes | yes | yes | no | yes | yes | yes | yes | yes | no | yes | 10 |
| De Angelis (2025) [34] | yes | yes | yes | yes | yes | yes | yes | yes | yes | yes | yes | yes | 12 |
| De Freitas Costa (2024) [38] | yes | yes | yes | yes | yes | yes | yes | yes | yes | no | yes | yes | 11 |
| Fesce  (2023) [37] | yes | no | yes | no | yes | yes | yes | yes | yes | yes | yes | yes | 10 |
| Fornasiero  (2020) [51] | yes | yes | yes | yes | yes | yes | yes | yes | yes | yes | yes | yes | 12 |
| Groen  (2017) [45] | yes | yes | yes | no | yes | no | yes | yes | yes | no | yes | no | 8 |
| Jian  (2014) [52] | yes | no | yes | yes | yes | yes | yes | yes | yes | no | yes | yes | 10 |
| Marcantonio  (2015) [32] | yes | yes | yes | yes | yes | yes | no | yes | yes | yes | yes | yes | 11 |
| Marini  (2016) [47] | yes | no | yes | yes | yes | no | yes | yes | yes | yes | yes | yes | 10 |
| Marini  (2017) [46] | yes | yes | no | yes | yes | yes | yes | yes | yes | no | yes | yes | 10 |
| Marini  (2018) [41] | yes | yes | yes | yes | yes | yes | yes | no | yes | no | no | yes | 9 |
| Marini  (2020) [36] | yes | no | yes | yes | yes | no | yes | yes | yes | yes | yes | no | 9 |
| Marini  (2021) [28] | yes | no | yes | yes | yes | yes | yes | yes | yes | no | yes | yes | 10 |
| Marini (2022) [40] | yes | yes | yes | yes | yes | no | no | no | yes | no | yes | yes | 8 |
| Moirano  (2018) [33] | yes | yes | yes | yes | yes | yes | no | yes | yes | yes | yes | yes | 11 |
| Moirano (2025) [35] | yes | no | yes | no | yes | yes | yes | yes | yes | yes | yes | yes | 10 |
| Mughini-Gras  (2013) [30] | yes | yes | yes | no | yes | no | yes | yes | yes | yes | yes | yes | 10 |
| Mulatti  (2011) [31] | yes | no | yes | yes | no | yes | yes | yes | no | yes | no | yes | 8 |
| Mulatti  (2014) [55] | yes | no | yes | yes | yes | yes | yes | yes | yes | yes | no | yes | 10 |
| Rizzoli  (2015) [48] | yes | yes | yes | yes | yes | yes | yes | yes | yes | yes | yes | yes | 12 |
| Roiz  (2012) [49] | yes | yes | yes | yes | yes | yes | yes | yes | yes | no | yes | yes | 11 |
| Rosà  (2014) [50] | yes | yes | yes | yes | no | yes | yes | yes | yes | yes | yes | yes | 11 |
| Serres (2024) [42] | yes | yes | yes | yes | yes | yes | yes | yes | yes | yes | yes | yes | 12 |
| Trajer  (2017) [29] | yes | yes | yes | yes | yes | yes | yes | yes | no | yes | yes | no | 10 |

**Supplementary Table S3.** Overview of investigated factors in each included study (systematic review, Italy, 2011-2025).

| **First author (year) [reference number]** | **Analytical approach*** | **Temperature** | **Precipitation** | **Humidity** | **Wind** | **ET** | **Daytime length, season** | **Other** | **Vegetation/ NDVI/EVI**** | **Standing water bodies** | **NDWI *** (water index)** | **Altitude, slope** | **Land use** | **Animal dynamics** | **Vector density** | **Distance to specific areas** | **Human population density** | **Animal host density** | **Other (*Aedes* competition, Aridity)** |
| --- | --- | --- | --- | --- | --- | --- | --- | --- | --- | --- | --- | --- | --- | --- | --- | --- | --- | --- | --- |
| Bisanzio  (2011) [43] | SM/ AM | X | X |  |  |  |  |  | X |  |  | X | X |  |  | X |  |  |  |
| Calzolari  (2015) [54] | SM | X | X |  |  |  |  |  |  |  |  |  |  |  | X |  |  |  |  |
| Calzolari  (2020) [53] | SM/ DM | X | X |  |  | X |  |  | X |  |  |  |  |  |  |  |  |  |  |
| Candeloro  (2020) [39] | PM | X |  |  |  |  |  |  | X |  |  |  |  |  |  | X |  |  |  |
| Carrieri  (2014) [44] | AM | X | X | X | X | X |  | X |  |  |  |  |  |  | X |  |  |  |  |
| Conte  (2015) [56] | AM/ PM | X |  |  |  |  |  |  | X |  |  | X |  |  |  |  |  |  |  |
| De Angelis (2025) [34] | AM | X | X | X |  |  | X |  |  |  |  |  |  |  |  |  |  |  |  |
| De Freitas Costa (2024) [38] | TM/ DM | X |  |  |  |  |  |  |  |  |  |  |  |  | X |  |  |  | X |
| Fesce  (2023) [37] | TM/ PM | X |  |  |  |  |  |  |  |  |  |  |  |  |  |  |  |  | X |
| Fornasiero  (2020) [51] | AM | X | X |  |  |  | X |  | X |  |  |  |  |  | X |  |  |  | X |
| Groen  (2017) [45] | PM | X | X | X |  |  |  |  |  |  |  |  |  |  |  |  |  |  |  |
| Jian  (2014) [52] | AM | X | X |  | X |  | X |  | X |  |  |  |  |  |  | X |  |  |  |
| Marcantonio  (2015) [32] | AM/ PM | X | X |  |  |  | X |  | X | X | X |  | X |  |  |  |  |  |  |
| Marini  (2016) [47] | DM | X | X |  |  |  |  |  |  |  |  |  |  |  | X |  |  |  |  |
| Marini  (2017) [46] | DM | X | X |  |  |  |  |  |  |  |  |  |  |  |  |  |  |  | X |
| Marini  (2018) [41] | DM/ TM |  |  |  |  |  | X |  |  |  |  |  |  |  |  |  |  |  |  |
| Marini  (2020) [36] | TM/ AM | X |  |  |  |  |  |  |  |  |  |  |  |  | X |  |  |  |  |
| Marini  (2021) [28] | AM | X |  |  |  |  |  |  |  |  |  |  |  |  |  |  |  |  |  |
| Marini (2022) [40] | AM | X | X |  |  |  |  |  |  |  |  |  | X |  |  | X | X |  | X |
| Moirano  (2018) [33] | AM | X | X |  |  |  |  |  |  |  |  |  |  |  |  |  |  |  |  |
| Moirano (2025) [35] | AM | X | X |  |  |  |  |  |  |  |  |  |  |  |  |  |  |  |  |
| Mughini-Gras  (2013) [30] | PM | X | X |  |  |  |  |  | X | X |  | X | X |  |  |  | X | X |  |
| Mulatti  (2011) [31] | AM | X | X |  |  |  |  |  | X |  |  | X |  |  |  |  |  |  |  |
| Mulatti  (2014) [55] | AM | X | X | X |  |  | X |  |  |  |  |  |  |  | X |  |  |  |  |
| Rizzoli  (2015) [48] | AM |  |  |  |  |  |  |  |  |  |  |  |  | X |  |  |  |  |  |
| Roiz  (2012) [49] | AM | X | X |  |  |  |  |  |  |  |  |  |  |  |  |  |  |  |  |
| Rosà  (2014) [50] | AM | X | X |  |  |  |  |  |  |  | X |  |  |  |  | X |  |  |  |
| Serres (2024) [42] | PM | X | X | X |  |  |  |  | X | X |  |  | X |  |  | X |  |  |  |
| Trajer  (2017) [29] | AM | X | X |  |  |  |  |  |  |  |  |  |  |  |  |  |  |  |  |

*Analytical approach: predictive models (PM), association models (AM), density models (DM), spatial models (SM), transmission models (TM). **NDVI/EVI: normalized difference vegetation index/Landsat enhanced vegetation index. *** NDWI: normalized difference water index

**Supplementary Table S4.** Details of data sources for climatic and environmental factors (systematic review, Italy, 2011-2025).

| **First author (year)** | **Type of maps* (legenda): PM, AM** | **Analysed region** | **Local registers** | **MODIS (NASA)** | **Copernicus** | **WorldClim** | **ECA&D** | **ARPA** | **Others** |
| --- | --- | --- | --- | --- | --- | --- | --- | --- | --- |
| Bisanzio  (2011) [43] | AM, PM | Piedmont |  | X |  |  |  | X |  |
| Calzolari  (2015) [54] | AM | Emilia-Romagna and Lombardia |  |  |  |  | X |  |  |
| Calzolari  (2020) [53] | AM | Padan Plain |  | X |  |  |  | X | Vecmap, EUMETSAT |
| Candeloro  (2020) [39] | PM | Italy |  | X | X |  |  |  |  |
| Carrieri  (2014) [44] |  | Bologna Province | X |  |  |  |  |  |  |
| Conte  (2015) [56] | PM | Italy |  | X |  |  |  |  | Global 30-Arc-Second Elevation Dataset for theWorld, ArcGIS10 |
| De Angelis (2025) [34] | AM | Northern Italy |  |  |  |  |  |  | ISPRA (SCIA), ISTAT |
| De Freitas Costa [2024] (38) |  | Italy |  |  | X |  |  |  |  |
| Fesce  (2023) [37] |  | Lombardy |  |  |  |  |  | X |  |
| Fornasiero  (2020) [51] |  | Veneto, FVG** |  | X |  |  |  | X |  |
| Groen  (2017) [45] |  | Emilia-Romagna, River Po Delta |  |  |  |  |  | X |  |
| Jian  (2014) [52] |  | Rovigo Province |  |  |  |  |  | X | EEA, ASTER, SPOT 5, DEM |
| Marcantonio  (2015) [32] | AM | All Europe |  | X |  |  | X |  | Globcover, Anthromes, IUCN and UNEP |
| Marini  (2016) [47] |  | Eastern Piedmont Region |  |  |  |  |  | X | US Naval Observatory |
| Marini  (2017) [46] |  | Trento and Belluno Province |  | X |  |  |  |  |  |
| Marini  (2018) [41] |  | Veneto |  |  |  |  |  | X |  |
| Marini  (2020) [36] | AM | Emilia-Romagna |  |  |  |  |  |  | ND |
| Marini  (2021) [28] | AM | All Europe |  | X |  |  |  |  |  |
| Marini (2022) [40] | AM | All Europe |  | X |  |  |  |  | Climate Data Store,  Corine Land Cover (CLC), NUTS3 |
| Moirano  (2018) [33] | AM | Northern Italy |  |  |  |  |  | X |  |
| Moirano (2025) [35] | AM | All Europe |  |  |  |  |  |  | ERA5 |
| Mughini-Gras  (2013) [30] | AM, PM | Italy |  |  |  | X |  |  | Corine Land Cover (CLC), ISTAT |
| Mulatti  (2011) [31] | PM | Padua, Rovigo and Venice provinces |  |  |  |  |  |  | ND |
| Mulatti  (2014) [55] | AM | Veneto, FVG** |  |  |  |  |  |  | Field data |
| Rizzoli  (2015) [48] |  | Veneto |  |  |  |  |  |  | Field data |
| Roiz  (2012) [49] |  | Trentino (Comune di Arco e Riva del Garda) | X |  |  |  |  |  |  |
| Rosà  (2014) [50] |  | Piedmont |  |  |  |  | X |  |  |
| Serres (2024) [42] | PM | All Europe |  |  |  |  |  |  | Inter-Sectoral Impact Model Inter-comparison Project (ISIMIP), ERA5, Corine Land Cover |
| Trajer  (2017) [29] |  | Italy |  |  |  |  | X |  | KNMI Climate Explorer |

*Type of maps: abundance map (AM), predictive map (PM). ******FVG: Friuli Venezia Giulia
